# Diffusion Tensor Imaging in NAWM and NADGM in MS and CIS: Association with Candidate Biomarkers in Sera

**DOI:** 10.1155/2013/265259

**Published:** 2013-12-17

**Authors:** Renuka Natarajan, Sanna Hagman, Xingchen Wu, Ullamari Hakulinen, Minna Raunio, Mika Helminen, Maija Rossi, Prasun Dastidar, Irina Elovaara

**Affiliations:** ^1^Neuroimmunology Unit, Medical School, University of Tampere, 33520 Tampere, Finland; ^2^Department of Neurology, Tampere University Hospital, 33520 Tampere, Finland; ^3^Department of Radiology, Medical Imaging Centre, Tampere University Hospital, 33520 Tampere, Finland; ^4^Department of Neurology, Seinäjoki Central Hospital, 60220 Seinäjoki, Finland; ^5^Science Center, Pirkanmaa Hospital District and School of Health Sciences, University of Tampere, 33520 Tampere, Finland; ^6^Department of Electronics and Communications Engineering, Tampere University of Technology, 33520 Tampere, Finland

## Abstract

The aim of this study was to evaluate diffusion tensor imaging (DTI) indices in the corpus callosum and pyramidal tract in normal-appearing white matter (NAWM) and the caudate nucleus and thalamus in deep grey matter (NADGM) in all MS subtypes and clinically isolated syndrome (CIS). Furthermore, it was determined whether these metrics are associated with clinical measures and the serum levels of candidate immune biomarkers. Apparent diffusion coefficients (ADC) values were significantly higher than in controls in all six studied NAWM regions in SPMS, 4/6 regions in RRMS and PPMS and 2/6 regions in CIS. In contrast, decreased fractional anisotropy (FA) values in comparison to controls were detected in 2/6 NAWM regions in SPMS and 1/6 in RRMS and PPMS. In RRMS, the level of neurological disability correlated with thalamic FA values (*r* = 0.479, *P* = 0.004). In chronic progressive subtypes and CIS, ADC values of NAWM and NADGM were associated with the levels of MIF, sFas, and sTNF-**α**. Our data indicate that DTI may be useful in detecting pathological changes in NAWM and NADGM in MS patients and that these changes are related to neurological disability.

## 1. Introduction

Multiple sclerosis (MS) is the most common autoimmune disease of the central nervous system (CNS), and it is characterised by inflammation, demyelination and degenerative changes [1]. The identification of surrogate markers reflecting pathophysiological events in the CNS and correlating with clinical outcomes is highly needed for refining diagnostics and developing therapeutic approaches in patients with MS [2, 3]. Magnetic resonance imaging (MRI) is the most valuable paraclinical tool for monitoring the disease process *in vivo*, but the correlations between clinical and conventional imaging measures detected thus far have been generally suboptimal [4]. This phenomenon is most likely explained by the limitations of expanded disability status scale (EDSS) scoring and the ability of conventional MRI to reflect changes in the CNS consistent with different manifestations of MS [4, 5].

Recent neuropathological studies in MS have shown widespread tissue damage in both normal-appearing white matter (NAWM) and grey matter (NAGM) tissues [6] that are not detected by conventional MRI [7]. Nonconventional MRI approaches such as diffusion tensor imaging (DTI) allow for further examination of brain tissues *in vivo*. DTI utilises the orientation-dependent diffusion property of water molecules within the CNS and provides unique information on the pathological processes that reflect the microstructural damage in brain [8]. Tissue changes studied with DTI are measured by fractional anisotropy (FA) and apparent diffusion coefficient (ADC). Most of the studies on NAWM in MS have reported increased ADC and reduced FA when compared to corresponding white matter regions in healthy subjects [9–11]. It is believed that changes in these measures reflect axonal damage and demyelination as well as inflammatory processes [12], although the evidence of corresponding pathological alterations is less clear than that in conventional MRI. Recent studies on NAGM emphasize that its damage occurs from the earliest stages of the disease process and may be a major determinant of long-term outcomes in MS [13–15]. However, attempts to correlate DTI indices with clinical measures have provided conflicting results [16–23].

The identification of biomarkers that could indicate pathophysiological processes or responses to a therapeutic intervention in individual MS patients would facilitate both diagnostic approaches and selection of treatments. So far, several candidate biomarkers have been identified in blood and cerebrospinal fluid (CSF) of MS patients [24, 25]. Among these biomarkers, several molecules, including cytokines, chemokines, adhesion molecules, antibodies, and apoptotic proteins, have been studied by us and others, and some have been associated with disease activity, neurological disability, or therapeutic responses [25–29]. However, the correlations between these molecules and conventional MRI findings have been relatively weak [30, 31]. In this study, our aim was to assess whether DTI indices in NAWM and normal-appearing deep grey matter (NADGM) are associated with different subtypes of MS and CIS and to address their association with clinical measures and the levels of candidate immune biomarkers in sera.

## 2. Patients and Methods

### 2.1. Study Population

We studied a total of 110 patients (75 females and 35 males), including patients with relapsing remitting MS (RRMS, *n* = 36), secondary progressive MS (SPMS, *n* = 19), primary progressive MS (PPMS, *n* = 21), clinically isolated syndrome (CIS, *n* = 24), and 10 healthy controls. All patients were followed up at the MS outpatient department at Tampere University Hospital and were recruited consecutively to this study. The study was approved by the Ethics Committee of Tampere University Hospital. All patients gave their informed consent. The diagnosis of MS was based on the revised McDonald criteria and CIS patients were defined as patients who had their first clinical episode suggestive of MS [32]. All RRMS and SPMS patients were in remission. We excluded the patients who were pregnant or suffering from any other clinically significant disease or treated with immunosuppressive drugs at least eight weeks before entering the study. Patients underwent neurological and MRI examinations as well as blood sampling on the same day. The determination of neurological disability was based on the EDSS score [33], and the disease activity was based on the number of relapses in the two years before study entry. All the clinical characteristics and immunomodulatory treatments are summarised in Table 1.

### 2.2. MR Image Acquisition

MR imaging was acquired using a 1.5-Tesla MR scanner (Siemens Avanto, Erlangen, Germany). All of the subjects were examined using the same MRI protocol, which consisted of a sagittal T1-weighted three-dimensional (3D) inversion recovery (IR)prepared gradientecho imaging, an axial T2-weighted turbo spin-echo imaging, a conventional axial and a high resolution sagittal fluid attenuation inversion recovery (FLAIR) imaging, an axial T2*-weighted imaging, and an axial susceptibility-weighted imaging (SWI). The DTI data were collected by a single-shot spin-echo-based echo-planar diffusion-weighted imaging (EPI) sequence with the following parameters: repetition time (TR) 3500 milliseconds (ms), echo time (TE) 96 ms, slice thickness 5 mm, interslice gap 1.5 mm, field of view (FOV) 230 mm, matrix 128 × 128 (in-plane resolution = 1.8 × 1.8 mm^2^), *b* values 0 and 1000 s/mm^2^, number of excitations 3, and with 12 diffusion gradient orientations. The total scanning time was approximately 30 minutes.

For the volumetric analysis of T2-weighted plaques we used FLAIR sequence. The parameters used in this sequence are TR = 8500 ms; TE = 100 ms; TI = 2500 ms; slice thickness = 5.0 mm; in-plane resolution = 0.45∗0.45 mm.

### 2.3. MR Imaging Postprocessing and Analysis 

#### 2.3.1. DTI Analysis

**Figure 1:**
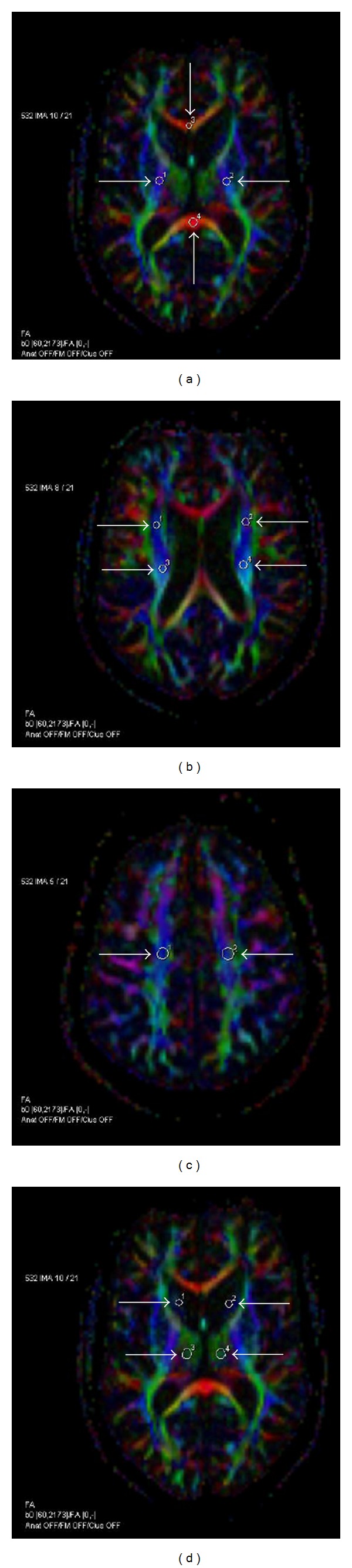
Region-of-interest (ROI) placement on axial FA colour maps. The posterior limb of the internal capsule (1, 2), the splenium (3), and the genu (4) of the corpus callosum (a); the posterior corona radiata anterior (1, 2) and posterior (3, 4) (b); the centrum semiovale anterior (c); and the caudate nucleus (1, 2) and the thalamus (3, 4) (d). Colours indicate the directions of fibre tracts (*red*, transverse; *blue*, craniocaudal; *green*, anterior-posterior). The circular ROIs were transferred from the corresponding *B*_0_ image, and their sizes have been adjusted to avoid any visible lesions. The size of the ROIs ranged from 2 to 33 pixels (6–106.5 mm^2^; pixel size 1.8∗1.8 mm^2^) depending on the size of the brain structure. This figure is a representative analysis from an SPMS patient.

The DTI analysis was performed as previously described [34] by an experienced radiologist (PD) together with a physicist (UH) who was blinded to the clinical details of the study subjects. The analysis was performed using the commercial software Neuro 3D (Siemens Healthcare, Malvern, PA, USA) on an offline workstation. In every individual, circular regions of interest (ROIs) of approximately 6 to 106 mm^2^ (depending on the anatomical regions) were manually placed simultaneously in exactly the same location on *B*_0_ images, ADC and FA maps. The ROIs were placed bilaterally (except for the corpus callosum) at the following anatomical locations: the posterior limb of the internal capsule, the centrum semiovale anterior, the posterior corona radiata anterior and posterior, the splenium and the genu of the corpus callosum, the thalamus, and the caudate nucleus (Figure 1). The ROIs were centred in the structure of interest in the most homogenous area, avoiding border areas to avoid the partial volume effects. The size of the ROI was reduced if a lesion was identified in the predefined ROI. ROIs of the same size were drawn in images of the healthy control subjects at the same anatomic locations as those of the patients.

#### 2.3.2. Volumetric Analysis

Volumetric segmentation of plaques in the brain was performed using semiautomatic software Anatomatic operating in a PC/Windows 95 environment and the images were analysed blindly.

### 2.4. Immunological Assay

The levels of cytokines, chemokines, and apoptotic molecules could be determined in 71 patients with MS (33RRMS, 18 SPMS, 20 PPMS), 15 subjects with CIS, and 21 controls in which blood sample was available. Sera were separated from blood and analysed for 14 different molecules: interleukin (IL)-2, IL-6, IL-10, IL-12p70, interferon (IFN)-*γ* macrophage migration inhibitory factor (MIF), tumor necrosis factor (TNF)-*α*, TNF-related apoptosis inducing ligand (TRAIL), sFas and Fas ligand (sFasL), and chemokines CXCL10, CCL2, CCL3, and CCL4 as previously described [26]. The levels of the molecules were determined by Luminex (Bio-Plex suspension array system, Bio-Rad laboratories, CA, USA). The levels of TRAIL were determined by ELISA.

### 2.5. Statistical Analysis

Assessments of clinical, DTI, and immunological data were analysed with PASW Statistics for Windows version 18.0 (SPSS Inc., Chicago, IL, USA). Average DTI values were calculated from the right and left sides to obtain a single value for each region. The differences in DTI indices between and within groups were assessed using the univariate analysis of variance with age as covariate followed by a post hoc multiple pairwise comparisons with Bonferroni's correction. Comparisons were considered to be statistically significant, if the *P* value was smaller than 0.005 (*n* = 10; 5 groups) or 0.0006 (*n* = 80; 5 groups and 8 brain regions) after Bonferroni's correction. Relationship between the DTI indices and clinical parameter (EDSS, number of relapses, disease duration, age) and immunological molecules MIF, sTNF-*α*, and sFas was studied with Spearman correlation coefficient. *P* values of correlation analyses were not corrected for multiple comparisons. In the correlation analyses, if *P* values smaller than 0.01 were considered to be statistically significant.

## 3. Results

### 3.1. Clinical Data

The clinical characteristics including disease duration, its prestudy activity, EDSS scores, and therapies of patients are summarised in Table 1. The RRMS patients had shorter disease duration and lower EDSS scores than patients with SPMS or PPMS. Two years before enrolment, seven of the 36 RRMS patients were relapse-free, 12 other patients had one relapse, and the remaining 17 subjects had 2 to 5 relapses. Twenty-one of 76 MS patients were treated with interferon-beta (IFN-*β*) and three other patients with glatiramer acetate. In the CIS group, the EDSS score was 0 except for three subjects with a score of 1.

### 3.2. Volumes of T1 and FLAIR Lesions

The volumes of FLAIR lesions were determined in the 73 patients with MS and 22 subjects with CIS. It appeared that in SPMS the volumes of FLAIR lesions were increased when compared to RRMS and CIS (RRMS: 8.0 ± 9.7; SPMS: 12.6 ± 9.3; PPMS: 9.0 ± 11.4; CIS: 2.1 ± 3.1 cm^3^; mean ± SD).

### 3.3. DTI Indices in Different Subtypes of MS and CIS

**Figure 2:**
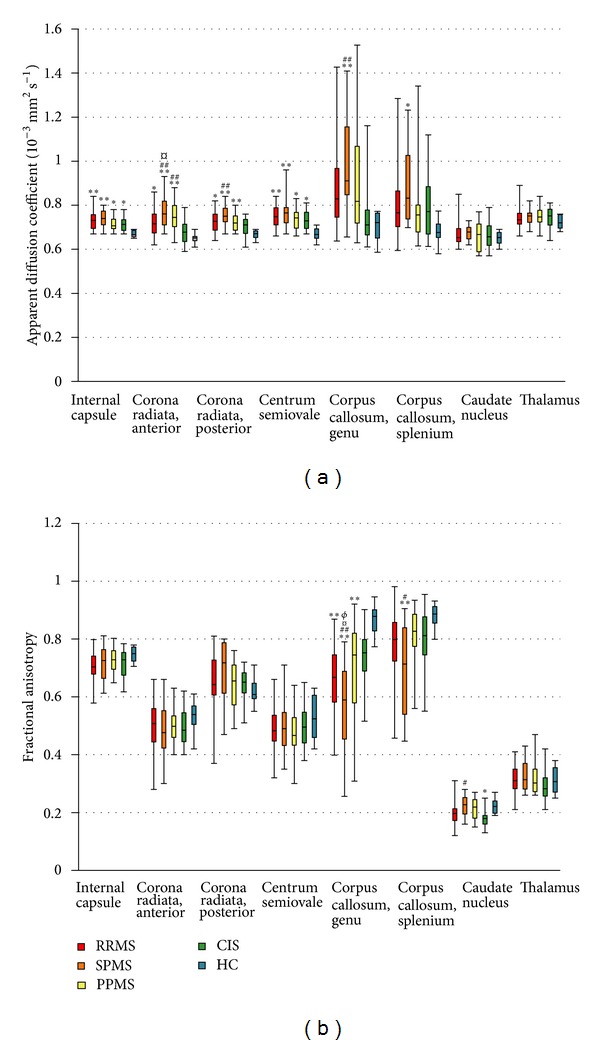
ADC and FA values in different brain regions of multiple sclerosis (MS) subtypes, clinically isolated syndrome (CIS), and healthy controls (HC). The length of the box represents the interquartile range, which includes the middle 50% of the values. The line through the middle of each box represents the median. The error bars show the minimum and maximum values (range). RRMS, relapsing remitting MS; SPMS, secondary progressive MS; PPMS, primary progressive MS; ADC, apparent diffusion coefficient; FA, fractional anisotropy. Reported *P* values were calculated using the univariate analysis of variance with age as covariate followed by post hoc multiple pairwise comparisons with Bonferroni correction. **P < 0.0006 (*n* = 80), *P < 0.005 (*n* = 10) when compared to HC. ^##^ *P* < 0.0006 (*n* = 80), ^#^ *P* < 0.005 (*n* = 10) when compared to CIS. ^¤^ *P* < 0.005  (*n* = 80) in comparison to RRMS. ^*∅*^ *P* < 0.005 (*n* = 80) in comparison to PPMS.

ADC and FA values of eight different anatomical brain regions were analysed from patients with different subtypes of MS, CIS, and healthy controls (Figure 2). Compared to controls, increased ADC values were detected in 6/8 regions in SPMS (internal capsule, corona radiata anterior and posterior, centrum semiovale, and splenium and genu of the corpus callosum), 4/8 regions in both RRMS or PPMS (internal capsule, corona radiata anterior and posterior, and centrum semiovale), and 2/8 regions (internal capsule and centrum semiovale) in CIS (Figure 2(a)). The corresponding comparison between FA indices showed significantly lower FA values in 2/8 regions (genu and splenium of corpus callosum) in SPMS, 1/8 regions in both RRMS and PPMS (genu of corpus callosum), and 1/8 regions (caudate nucleus) in CIS (Figure 2(b)).

Comparison between patients with different MS subtypes and CIS revealed higher ADC values in 3/8 regions (corona radiata anterior and posterior and genu of corpus callosum) in SPMS and in 1/8 regions (corona radiata anterior) in PPMS, but no differences were found between RRMS and CIS (*P* > 0.005, Figure 2(a)). Further comparisons between MS subtypes showed increased ADC values in 1/8 regions (corona radiata anterior) in SPMS compared to RRMS, but no differences were seen between RRMS or SPMS and PPMS (*P* > 0.005). Corresponding comparisons between FA values showed significantly lower FA values in 3/8 regions (splenium and genu of corpus callosum and caudate nucleus) of the SPMS group when compared to CIS, but no differences were found between CIS and RRMS or PPMS. (Figure 2(b)). Comparison between MS subtypes showed significantly lower FA values in one region (genu of the corpus callosum) in SPMS. No differences were found in FA between RRMS and PPMS.

### 3.4. Association between DTI Indices, Clinical Parameters, and Immune Molecules

**Figure 3:**
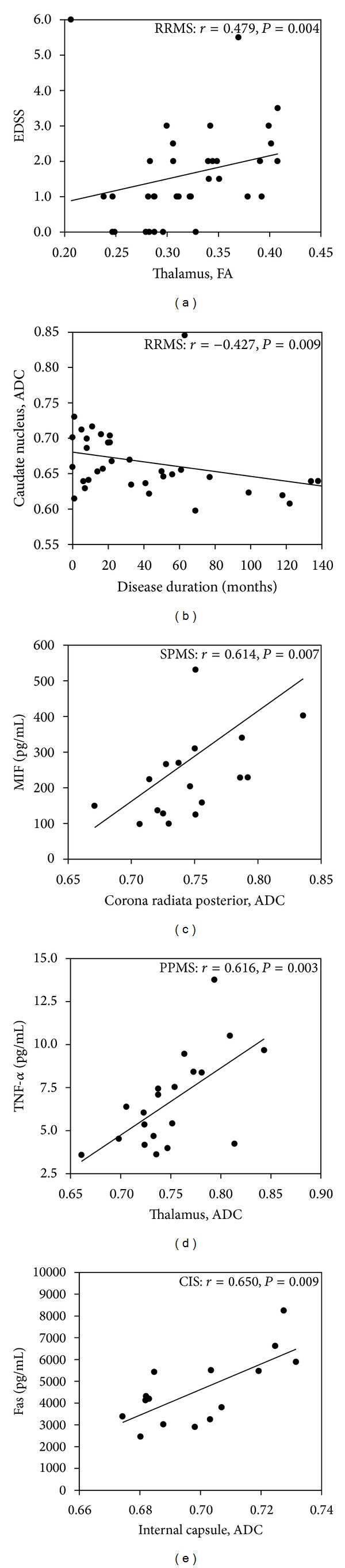
Correlations between DTI indices and clinical and immunological parameters in MS and CIS. Statistically significant correlations were found between the EDSS score and FA values for the thalamus in RRMS (a), disease duration and ADC values for the caudate nucleus in RRMS (b), the levels of MIF and the ADC values for the corona radiata posterior in SPMS (c), the levels of TNF-*α* and the ADC values for the thalamus in PPMS (d), and the levels of Fas and the ADC values for the internal capsule in CIS (e).

The ADC and FA values of the eight brain regions were further correlated with clinical parameters, including prestudy disease activity, EDSS scores, disease duration, age, and candidate immune biomarkers (Table 2 and Figure 3). According to clinicoradiological correlation analyses, the EDSS score of RRMS group correlated with its FA values of the thalamus (*r* = 0.479, *P* = 0.004, Figure 3(a)). The disease duration of RRMS group correlated with ADC value of caudate nucleus (*r* = −0.427, *P* = 0.009, Figure 3(b)), but no associations were found between the prestudy activity, age, and DTI indices in any of the groups.

The data on candidate immune biomarkers used in the correlation analyses of this study have been previously reported by our group (see Supplementary Table 1 in Supplementary Material available online at http://dx.doi.org/10.1155/2013/265259). In this study, we focused on those molecules (sFas, sTNF-*α*, and MIF) that appeared to be significant in our previous studies [26, 35] and were therefore considered as candidate biomarkers. The significant correlations between the DTI indices and biomarkers were restricted to the chronic progressive groups and CIS (Figures 3(c)–3(e)). In SPMS, the ADC values of the corona radiata posterior correlated with the levels of MIF (*r* = 0.614, *P* = 0.007, Figure 3(c)), while in PPMS, the ADC values of the thalamus correlated with sTNF-*α* (*r* = 0.616, *P* = 0.003, Figure 3(d)). In CIS, the ADC values of internal capsule correlated with the levels of sFas (*r* = 0.650, *P* = 0.009, Figure 3(e)).

## 4. Discussion

DTI is a promising technique for detecting demyelination and axonal loss in MS lesions and revealing diffuse microscopic changes in NAWM and NAGM. Until now, most of the previous DTI studies have been focused on particular MS subtypes [17, 23, 36, 37] or combined MS groups [10, 20], while only a few studies have included all MS subtypes and CIS [11, 38, 39]. Therefore, DTI studies covering the whole clinical spectrum of MS and CIS are required for evaluating the applicability of this methodology in clinical practice. In the present study, our purpose was to examine whether the DTI indices in the pyramidal tract and corpus callosum of NAWM and the caudate nucleus and thalamus of NADGM are associated with different subtypes of MS and CIS. Furthermore, we examined whether these measures are associated with neurological dysfunction and disease activity. We also studied the association between DTI indices and candidate immune biomarkers in sera.

According to this study, increased ADC values in NAWM regions are already present at the CIS stage, and these changes become most prevalent in SPMS. In the previous studies, DTI abnormalities have been detected in all MS subtypes and CIS, although the degree of damage differs between the subtypes [10, 11, 38–44]. In line with our data, Preziosa et al. showed increased ADC values in all studied NAWM regions beginning in the CIS stage, while the most pronounced microstructural damage was detected in SPMS [39]. FA abnormality was detected only in the corpus callosum regions, and it was most apparent in the SPMS phase. Similar results have been reported also by other investigators [11, 38, 43]. Cercignani et al. have detected lower FA and higher ADC values in the corpus callosum of patients with SPMS compared to patients with RRMS and PPMS [11]. Another study showed increased ADC values in the genu of the corpus callosum in SPMS when compared to controls and RRMS, but the FA values showed no significant differences [38]. Hannoun et al. measured DTI indices in the centrum semiovale and found both decreased FA values and increased ADC values in SPMS when compared to RRMS [43]. Thus, the data generated by us and others suggest that the greater increase in diffusivity is consistent with the more advanced phases of the disease, in which degenerative changes prevail over inflammation. Neuropathological studies have also shown that white matter is more severely affected in SPMS than in RRMS and PPMS [45]. In these studies, new and active white matter lesions are mainly detected in patients with RRMS, while diffuse inflammatory damage in NAWM and NAGM together with cortical demyelination is the hallmarks of chronic progressive subtypes [6]. Based on our data, it is noteworthy that diffusivity changes are present also in PPMS, but the damage in this subtype appears to be less severe than in SPMS. In respect to methodology, the data from our work and others' suggest that ADC indices are more sensitive than FA measures in detecting microstructural changes in the NAWM in MS. The present study applied an ROI-based approach, while other studies have also used histogram analyses detecting whole-brain damage [9, 40] and voxel-wise analysis detecting regional abnormalities [10, 39, 42, 46] in parallel. We consider that DTI may be useful in detecting pathological processes in MS and might prove valuable in clinical practice.

In RRMS, a strong association was detected between the FA values of thalamus and neurological disability expressed by EDSS score. This is in line with earlier studies reporting a positive correlation between the FA and ADC values of the thalamus and caudate nucleus and neurological disability in RRMS [47] and SPMS [47, 48]. These studies have additionally reported higher ADC and FA values in the caudate nucleus and thalamus in these subtypes compared to healthy controls, although in the present study, such differences between the subtypes and controls could not be found. It is suggested that increased FA in grey matter might indicate microglial activation or other inflammatory events [49]. Moreover, the conventional MRI studies have shown the presence of atrophy of the caudate nucleus and thalamus even in the earliest stages of the disease [13, 50]. Such thalamic damage in MS patients has been associated with physical disability and cognitive impairment [51, 52]. Thus, based on these data, the DTI indices of NADGM may be useful indicators of disability accumulation in early MS.

Correlation between DTI findings and immunological molecules is a new approach for identifying biomarkers that could be useful both in refining the diagnostics and in selecting and optimising the appropriate MS therapy. Thus far, the correlations between candidate immune markers and lesion volumes quantified by conventional MRI have been relatively weak [30, 31]. Neuropathological studies have shown that mild inflammation, along with microglial activation, gliosis, diffuse axonal injury, and nerve fibres degeneration, is present also in NAWM and NAGM [53]. Therefore, our aim was to explore whether the proinflammatory molecules MIF, sFas, and TNF-*α*, earlier considered [26, 35] to be important in MS, would be associated with the diffusion and anisotropic changes in NAWM and NADGM. It is generally considered that immunological markers in blood at least partially reflect the inflammatory activity within the blood-brain-barrier compartment [54]. CSF would be better for these purposes, but there are well-known limitations to its availability, and blood is therefore a good alternative for identifying biomarkers [55]. Recently, new and rapidly emerging technologies allowing large-scale identification of potential biomarkers have been developed, and they will provide new opportunities for biomarker discovery [56].

In our previous study, we showed that increased MIF levels are associated with clinical disease activity in RRMS [26, 35], while increased levels of sTNF-*α* and sFas in sera in PPMS indicate the presence of inflammatory activity in this subtype [26]. MIF is released from its cytoplasmic stores during immune activation, and it promotes the migration of inflammatory cells into the CNS [57]. sTNF-*α* mediates apoptosis of oligodendrocytes and promotes inflammation [58], while sFas is shown to inhibit Fas-mediated apoptosis [59]. In this study, the levels of blood inflammatory molecules obtained from 86 out of 100 patients were correlated with DTI indices. In patients with chronic progressive subtypes and CIS in the present study, these molecules correlated with diffusivity changes in the corona radiata posterior and internal capsule of NAWM and the thalamus of NADGM (Figures 3(c)–3(e)). Thus, these observations and previous studies suggest that these molecules, which reflect inflammatory disease activity, are associated with development of microstructural changes in MS.

## 5. Conclusions

The present study showed that the most abnormal DTI indices were present in SPMS, although changes were seen throughout the spectrum of MS disease and CIS. The association between disability and the thalamic FA indices suggests that DTI might be a useful indicator of neurological disability. However, determining the utility of this methodology will require longitudinal studies. The association between the DTI indices in both NADGM and NAWM and the levels of MIF, sFas, and sTNF-*α* suggests the involvement of these molecules in promoting such microstructural changes in the CNS.

## Supplementary Material

The levels of cytokines, chemokines and apoptotic molecules could be determined in 71 patients with MS and 15 subjects with CIS and 21 healthy controls. The supplementary material presents the levels of soluble molecules in different subtypes of MS and controls and the clinical characteristics of these patients

## Figures and Tables

**Table 1:** Clinical characteristics of patients with different subtypes of multiple sclerosis, clinically isolated syndrome, and controls.

Clinical characteristic	Patients with MS and CIS (*n* = 100)				
	RRMS *n* = 36	SPMS *n* = 19	PPMS *n* = 21	CIS *n* = 24	HC *n* = 10
Sex (M/F)^a^	11/25	7/12	9/12	3/21	4/6
Age (years)^b^	36.6 ± 8.4 (18–53)	49.5 ± 8.2^B,C^ (35–61)	57.0 ± 9.2^A,B,C,D^ (38–73)	34.3 ± 9.5 (20–52)	39.8 ± 12.9 (26–61)^A^
Duration of disease (years)^b^	3.9 ± 3.9 (0.0–12.3)	11.3 ± 9.3^C^ (0.2–31.2)	11.9 ± 8.4^C^ (0.2–26.2)	NA	NA
EDSS^b^	1.7 ± 1.6^B^ (0–6)	4.7 ± 1.7^B,C^ (2–7)	4.8 ± 2.0^B,C^ (1–8)	0.0 ± 0.2 (0–1)	NA
Number of relapses/2 years^b,c^	1.6 ± 1.4 (0–5)	0.2 ± 0.5 (0–2)	NA	0.8 ± 0.6 (0–2)	NA
Treatment (NT/IFN/GA)^a^	13/20/3	18/1/0	21/0/0	24/0/0	NA

MS: multiple sclerosis; CIS: clinically isolated syndrome; RRMS: relapsing-remitting MS; SPMS: secondary progressive MS; PPMS: primary progressive MS; HC: healthy controls; EDSS: expanded disability status scale; NT: no treatment; IFN: interferon-*β*; GA: glatiramer acetate.

^a^Number of patients.

^b^Mean ± SD (range).

^c^Number of relapses in the two years before study entry.

Analyses were performed for all four subtypes versus control, as well as between the subtypes. The results from the Mann-Whitney *U* test are indicated as the Bonferroni-corrected *P*-values (*P* < 0.05).

^A^compared to controls.

^B^compared to CIS.

^C^compared to RRMS.

^D^compared to SPMS.

**Table 2:** Clinicoradiological correlations between DTI indices and clinical findings in RRMS patients (*n* = 35).

	EDSS	Relapses^a^	Age	DD
Internal capsule				
ADC	−0.094	−0.149	−0.078	−0.072
FA	0.310	−0.128	−0.143	0.200
Corona radiata, posterior				
ADC	0.099	0.002	0.083	0.080
FA	0.349	−0.032	0.151	0.381
Corona radiata, anterior				
ADC	0.200	−0.177	0.036	0.055
FA	0.359	−0.071	−0.062	0.307
Centrum semiovale				
ADC	0.027	−0.021	0.155	0.149
FA	0.122	0.096	−0.057	0.162
Corpus callosum, genu				
ADC	0.061	−0.277	0.133	−0.002
FA	−0.224	0.153	−0.221	0.002
Corpus callosum, splenium				
ADC	0.028	0.277	−0.368	0.051
FA	−0.115	−0.017	0.175	0.073
Thalamus				
ADC	−0.339	−0.110	−0.323	−0.332
FA	0.479**	−0.304	0.384	0.292
Caudate nucleus				
ADC	−0.182	−0.146	−0.132	−0.427**
FA	0.390	0.058	0.264	0.139

EDSS: expanded disability status scale; DD: disease duration; ADC: apparent diffusion coefficient; FA: fractional anisotropy.

Significant *P* values: **P < 0.001.

^a^Number of relapses in the two years before study entry.

## References

[1] Wu GF, Alvarez E (2011). The immunopathophysiology of multiple sclerosis. *Neurologic Clinics*.

[2] Comabella M, Vandenbroeck K (2011). Pharmacogenomics and multiple sclerosis: moving toward individualized medicine. *Current Neurology and Neuroscience Reports*.

[3] Ziemann U, Wahl M, Hattingen E, Tumani H (2011). Development of biomarkers for multiple sclerosis as a neurodegenerative disorder. *Progress in Neurobiology*.

[4] Filippi M, Agosta F (2010). Imaging biomarkers in multiple sclerosis. *Journal of Magnetic Resonance Imaging*.

[5] Cohen JA, Reingold SC, Polman CH, Wolinsky JS (2012). Disability outcome measures in multiple sclerosis clinical trials: current status and future prospects. *The Lancet Neurology*.

[6] Kutzelnigg A, Lucchinetti CF, Stadelmann C (2005). Cortical demyelination and diffuse white matter injury in multiple sclerosis. *Brain*.

[7] Filippi M, Absinta M, Rocca MA (2013). Future MRI tools in multiple sclerosis. *Journal of the Neurological Sciences*.

[8] Rovaris M, Agosta F, Pagani E, Filippi M (2009). Diffusion tensor MR imaging. *Neuroimaging Clinics of North America*.

[9] Vrenken H, Geurts JJG, Knol DL (2006). Normal-appearing white matter changes vary with distance to lesions in multiple sclerosis. *American Journal of Neuroradiology*.

[10] Roosendaal SD, Geurts JJG, Vrenken H (2009). Regional DTI differences in multiple sclerosis patients. *NeuroImage*.

[11] Cercignani M, Bozzali M, Iannucci G, Comi G, Filippi M (2002). Intra-voxel and inter-voxel coherence in patients with multiple sclerosis assessed using diffusion tensor MRI. *Journal of Neurology*.

[12] Poloni G, Minagar A, Haacke EM, Zivadinov R (2011). Recent developments in imaging of multiple sclerosis. *Neurologist*.

[13] Henry RG, Shieh M, Amirbekian B, Chung S, Okuda DT, Pelletier D (2009). Connecting white matter injury and thalamic atrophy in clinically isolated syndromes. *Journal of the Neurological Sciences*.

[14] Tao G, Datta S, He R, Nelson F, Wolinsky JS, Narayana PA (2009). Deep gray matter atrophy in multiple sclerosis: a tensor based morphometry. *Journal of the Neurological Sciences*.

[15] Battaglini M, Giorgio A, Stromillo ML (2009). Voxel-wise assessment of progression of regional brain atrophy in relapsing-remitting multiple sclerosis. *Journal of the Neurological Sciences*.

[16] Griffin CM, Chard DT, Ciccarelli O (2001). Diffusion tensor imaging in early relapsing-remitting multiple sclerosis. *Multiple Sclerosis*.

[17] Hasan KM, Gupta RK, Santos RM, Wolinsky JS, Narayana PA (2005). Diffusion tensor fractional anisotropy of the normal-appearing seven segments of the corpus callosum in healthy adults and relapsing-remitting multiple sclerosis patients. *Journal of Magnetic Resonance Imaging*.

[18] Wilson M, Tench CR, Morgan PS, Blumhardt LD (2003). Pyramidal tract mapping by diffusion tensor magnetic resonance imaging in multiple sclerosis: improving correlations with disability. *Journal of Neurology Neurosurgery and Psychiatry*.

[19] Cader S, Johansen-Berg H, Wylezinska M (2007). Discordant white matter N-acetylasparate and diffusion MRI measures suggest that chronic metabolic dysfunction contributes to axonal pathology in multiple sclerosis. *NeuroImage*.

[20] Ciccarelli O, Werring DJ, Wheeler-Kingshott CAM (2001). Investigation of MS normal-appearing brain using diffusion tensor MRI with clinical correlations. *Neurology*.

[21] Lin X, Tench CR, Morgan PS, Niepel G, Constantinescu CS (2005). ’Importance sampling’ in MS: use of diffusion tensor tractography to quantify pathology related to specific impairment. *Journal of the Neurological Sciences*.

[22] Ciccarelli O, Wheeler-Kingshott CA, McLean MA (2007). Spinal cord spectroscopy and diffusion-based tractography to assess acute disability in multiple sclerosis. *Brain*.

[23] Giorgio A, Palace J, Johansen-Berg H (2010). Relationships of brain white matter microstructure with clinical and MR measures in relapsing-remitting multiple sclerosis. *Journal of Magnetic Resonance Imaging*.

[24] Derfuss T (2012). Personalized medicine in multiple sclerosis: hope or reality?. *BMC Medicine*.

[25] Pravica V, Markovic M, Cupic M (2013). Multiple sclerosis: individualized disease susceptibility and therapy response. *Biomarkers in Medicine*.

[26] Hagman S, Raunio M, Rossi M, Dastidar P, Elovaara I (2011). Disease-associated inflammatory biomarker profiles in blood in different subtypes of multiple sclerosis: prospective clinical and MRI follow-up study. *Journal of Neuroimmunology*.

[27] Ukkonen M, Wu X, Reipert B, Dastidar P, Elovaara I (2007). Cell surface adhesion molecules and cytokine profiles in primary progressive multiple sclerosis. *Multiple Sclerosis*.

[28] Tumani H, Hartung H-P, Hemmer B (2009). Cerebrospinal fluid biomarkers in multiple sclerosis. *Neurobiology of Disease*.

[29] Krumbholz M, Faber H, Steinmeyer F (2008). Interferon-*β* increases BAFF levels in multiple sclerosis: implications for B cell autoimmunity. *Brain*.

[30] Kraus J, Kuehne BS, Tofighi J (2002). Serum cytokine levels do not correlate with disease activity and severity assessed by brain MRI in multiple sclerosis. *Acta Neurologica Scandinavica*.

[31] Giovannoni G, Miller DH, Losseff NA (2001). Serum inflammatory markers and clinical/MRI markers of disease progression in multiple sclerosis. *Journal of Neurology*.

[32] Polman CH, Reingold SC, Edan G (2005). Diagnostic criteria for multiple sclerosis: 2005 revisions to the ‘McDonald criteria’. *Annals of Neurology*.

[33] Kurtzke JF (1983). Rating neurologic impairment in multiple sclerosis: an expanded disability status scale (EDSS). *Neurology*.

[34] Hakulinen U, Brander A, Ryymin P (2012). Repeatability and variation of region-of-interest methods using quantitative diffusion tensor MR imaging of the brain. *BMC Medical Imaging*.

[35] Rinta S, Kuusisto H, Raunio M (2008). Apoptosis-related molecules in blood in multiple sclerosis. *Journal of Neuroimmunology*.

[36] Tian W, Zhu T, Zhong J (2012). Progressive decline in fractional anisotropy on serial DTI examinations of the corpus callosum: a putative marker of disease activity and progression in SPMS. *Neuroradiology*.

[37] Sigal T, Shmuel M, Mark D, Gil H, Anat A (2012). Diffusion tensor imaging of corpus callosum integrity in multiple sclerosis: correlation with disease variables. *Journal of Neuroimaging*.

[38] Vrenken H, Pouwels PJW, Geurts JJG (2006). Altered diffusion tensor in multiple sclerosis normal-appearing brain tissue: cortical diffusion changes seem related to clinical deterioration. *Journal of Magnetic Resonance Imaging*.

[39] Preziosa P, Rocca MA, Mesaros S (2011). Intrinsic damage to the major white matter tracts in patients with different clinical phenotypes of multiple sclerosis: a voxelwise diffusion-tensor MR study. *Radiology*.

[40] Yu CS, Lin FC, Liu Y, Duan Y, Lei H, Li KC (2008). Histogram analysis of diffusion measures in clinically isolated syndromes and relapsing-remitting multiple sclerosis. *European Journal of Radiology*.

[41] Gallo A, Rovaris M, Riva R (2005). Diffusion-tensor magnetic resonance imaging detects normal-appearing white matter damage unrelated to short-term disease activity in patients at the earliest clinical stage of multiple sclerosis. *Archives of Neurology*.

[42] Raz E, Cercignani M, Sbardella E (2010). Clinically isolated syndrome suggestive of multiple sclerosis: voxelwise regional investigation of white and gray matter. *Radiology*.

[43] Hannoun S, Bagory M, Durand-Dubief F (2012). Correlation of diffusion and metabolic alterations in different clinical forms of multiple sclerosis. *PLoS One*.

[44] Tortorella P, Rocca MA, Mezzapesa DM (2006). MRI quantification of gray and white matter damage in patients with early-onset multiple sclerosis. *Journal of Neurology*.

[45] Bramow S, Frischer JM, Lassmann H (2010). Demyelination versus remyelination in progressive multiple sclerosis. *Brain*.

[46] Onu M, Roceanu A, Sboto-Frankenstein U (2012). Diffusion abnormality maps in demyelinating disease: correlations with clinical scores. *European Journal of Radiology*.

[47] Tovar-Moll F, Evangelou IE, Chiu AW (2009). Thalamic involvement and its impact on clinical disability in patients with multiple sclerosis: a diffusion tensor imaging study at 3T. *American Journal of Neuroradiology*.

[48] Hannoun S, Durand-Dubief F, Confavreux C (2012). Diffusion tensor-MRI evidence for extra-axonal neuronal degeneration in caudate and thalamic nuclei of patients with multiple sclerosis. *American Journal of Neuroradiology*.

[49] Calabrese M, Rinaldi F, Seppi D (2011). Cortical diffusion-tensor imaging abnormalities in multiple sclerosis: a 3-year longitudinal study. *Radiology*.

[50] Shiee N, Bazin PL, Zackowski KM (2012). Revisiting brain atrophy and its relationship to disability in multiple sclerosis. *PLoS One*.

[51] Mesaros S, Rocca MA, Pagani E (2011). Thalamic damage predicts the evolution of primary-progressive multiple sclerosis at 5 years. *American Journal of Neuroradiology*.

[52] Horakova D, Kalincik T, Blahova Dusankova J, Dolezal O (2012). Clinical correlates of grey matter pathology in multiple sclerosis. *BMC Neurology*.

[53] Lassmann H, Brück W, Lucchinetti CF (2007). The immunopathology of multiple sclerosis: an overview. *Brain Pathology*.

[54] Sospedra M, Martin R (2005). Immunology of multiple sclerosis. *Annual Review of Immunology*.

[55] Rajasekharan S, Bar-Or A (2012). From bench to MS bedside: challenges translating biomarker discovery to clinical practice. *Journal of Neuroimmunology*.

[56] Comabella M, Racke MK (2012). New technologies for biomarker discovery in multiple sclerosis. *Journal of Neuroimmunology*.

[57] Kithcart AP, Cox GM, Sielecki T (2010). A small-molecule inhibitor of macrophage migration inhibitory factor for the treatment of inflammatory disease. *The FASEB Journal*.

[58] Holtmann MH, Neurath MF (2004). Differential TNF-signaling in chronic inflammatory disorders. *Current Molecular Medicine*.

[59] Zipp F (2000). Apoptosis in multiple sclerosis. *Cell and Tissue Research*.

