# Supplementary material for: Diffusion Tensor Imaging in NAWM and NADGM in MS and CIS: Association with Candidate Biomarkers in Sera
Source: Mult Scler Int. 2013 Dec 17;2013:265259. doi: 10.1155/2013/265259 (PMC3877634; doi:10.1155/2013/265259)
Supplement: Supplementary file 1 — The levels of cytokines, chemokines and apoptotic molecules could be determined in 71 patients with MS and 15 subjects with CIS and 21 healthy controls. The supplementary material presents the levels of soluble molecules in different subtypes of MS and controls and the clinical characteristics of these patients [file 265259.f1.pdf]

Supplementary table 1. The levels (pg/ml) of soluble molecules in different subtypes of MS and controls (median (25-75th percentiles))

Molecule	RRMS n=33	SPMS n=18	PPMS n=20	CIS n=15	Controls n=21
**MIF**	138.2 (50.0-229.4) ^{A,C}	225.8 (134.0-317.3)	290.7 (189.3-449.3)^{C}	167.3 (97.8-268.2)	378.5 (236.4-535.9)^{A}
**sFas**	4612.8 (4058.0-5940.7)^{C}	5572.4 (4388.3-6845.4)	6787.6 (5393.9-7709.2) ^{B,C}	4318.8 (3320.0-5697.9) ^{G}	4824.9 (3405.1-5638.1) ^{B}
**sFasL**	90.6 (58.1-161.6)	96.2 (72.8-153.6)	111.2 (71.5-191.9)	83.6(67.1-174.6)	80.4 (56.6-129.1)
**sTRAIL**	765.9 (577.2-1728.9)	785.5 (587.2-1106.0)	1032.0 (762.8-2036.1)	902.3(593.3-1170.5)	738.7 (523.2-2260.8)
**sTNF-α**	4.5 (3.4-6.5)^{C}	4.1 (3.3-5.8)	6.4 (4.4-8.9) $^{B. C}$	4.6 (3.5-6.6)	3.7 (2.4-4.6) ^{B}
**IFN-γ**	0.3 (0.3-3.0)	1.5 (0.3-4.1)	0.6 (0.3-3.3)	2.8 (0.3-25.5)	0.5 (0.3-7.5)
**IL-2**	1.2 (0.2-3.6)	0.6 (0.2-5.2)	0.4 (0.2-2.4)	1.5 (0.5-6.2)	2.1 (0.2-6.1)
**IL-6**	4.9 (2.0-21.5)	7.1 (3.2-13.0)	8.8 (1.6-26.7)	3.6 (1.4-17.9)	4.1 (2.0-9.3)
**IL-10**	2.9 (0.6-5.7)	3.4 (1.8-7.7)	3.6 (2.2-9.2)	4.8 (1.2-25.9)	2.3 (0.2-6.0)
**IL-12p70**	0.4 (0.1-1.4)	1.3 (0.4-5.4)	0.8 (0.1-4.5)	1.7 (0.3-9.1)	1.6 (0.1-7.9)
**CXCL10**	95.7 (68.1-155.6)	90.5 (62.8-152.6)	106.9 (74.7-212.9)	65.9 (53.6-178.2)	106.9 (55.1-130.7)
**CCL2**	177.0 (140.4-271.8)	218.8 (157.0-331.0)	288.4 (201.3-461.6)^{B}	248.3 (165.7-332.0)	152.8 (122.6-273.1) ^{B}
**CCL3**	37.3 (21.6-96.3)	55.7 (19.2-121.4)	45.4 (27.0-164.7)	72.6 (32.0-186.1)	51.4 (27.7-122.8)
**CCL4**	126.6 (20.4-668.0)	140.2 (17.0-873.8)	290.7 (39.8-1054.6)	365.1 (144.0-1188.5)	289.1 (16.8-725.0)

RRMS, relapsing remitting MS; SPMS, secondary progressive MS; PPMS, Primary progressive MS; CIS, clinically isolated syndrome. Analyses were performed for all four subtypes versus control, as well as between the subtypes. The results from the Mann-Whitney U Test are shown as the Bonferroni-corrected p-values ($p<0.05$).

A Comparison between the RRMS and HC groups
B Comparison between the PPMS and HC groups
C Comparison between the PPMS and RRMS groups

Supplementary table 2. Clinical characteristics of patients and controls included in serum analyses.

	RRMS n=33	SPMS n=18	PPMS n=20	CIS n=15	HC n=21
Sex (M/F)[a]	10/23	6/12	9/11	2/13	8/13
Age (years) [b]	37.0±8.6 (18-53)	49.5±8.2 (35-61)	56.9±9.2 (38-73)	35.3±9.5 (20-52)	
Duration of disease (years) [b]	4.1±4.0 (0.0-12.3)	11.6±9.5 (0.2-31.2)	12.0±8.6 (0.2-26.2)	NA	
EDSS [b]	1.6±1.5 (0-6.0)	4.8±1.7 (2.0-7.0)	4.7±1.9 (1.0-7.5)	0.1±0.3 (0.0-1.0)	
Number of relapses/ 2years [b,c]	1.6±1.4 (0-5)	0.2±0.5 (0-2)	NA	0.8±0.6 (0-2)	
Treatment (NT/IFN/GA) [a]	12/18/3	17/1/0	20/0/0	15/0/0	

MS, multiple sclerosis; CIS, clinically isolated syndrome; RRMS, relapsing-remitting MS; SPMS, secondary progressive MS; PPMS, primary progressive MS; HC, healthy controls; EDSS, expanded disability status scale; NT, no treatment; IFN, interferon-β; GA, glatiramer acetate.

[a] Number of patients.

[b] Mean ± SD (range).

[c] Number of relapses in the two years before study entry
